# SUMOylation: an emerging field of protein modification in horticultural plants

**DOI:** 10.1093/hr/uhag194

**Published:** 2026-05-14

**Authors:** Chi Li, Xiaoshi Liu, Wenliang Li, Jianbin Lai

**Affiliations:** Guangdong Provincial Key Laboratory of Biotechnology for Plant Development, School of Life Sciences, South China Normal University, Guangzhou 510631, China; Guangdong Provincial Key Laboratory of Biotechnology for Plant Development, School of Life Sciences, South China Normal University, Guangzhou 510631, China; College of Life Science, Northwest Normal University, Lanzhou 730070, China; Guangdong Provincial Key Laboratory of Biotechnology for Plant Development, School of Life Sciences, South China Normal University, Guangzhou 510631, China

## Abstract

Horticultural plants play a vital role in agricultural production and daily life. Post-translational modifications are essential for regulating various cellular processes, but the functions of SUMOylation, an increasingly significant post-translational modification, remain to be thoroughly explored in horticultural plants. In this review, we summarize the current research advancements regarding protein SUMOylation in horticultural plants and offer perspectives for future investigations. We begin by introducing the SUMOylation system in horticultural plants. Subsequently, we focus on the roles of SUMOylation in development and stress responses in horticultural plants, with association of plant hormones in these processes. Following this discussion, we outline potential techniques for detecting SUMOylation substrates in horticultural species. Finally, we present prospective directions for future research in this field. This work aims to enhance our understanding of functional implications of SUMOylation while providing novel insights into strategies for improving yield, quality, and resistance in horticultural crops.

## A general introduction to SUMOylation

Horticultural plants, which encompass a diverse array of categories including fruits, vegetables, and ornamental species, hold substantial economic significance. These plants exhibit a remarkable degree of species diversity. Specifically, the categories of fruits, vegetables, and flowers comprise ~2800, 1000, and 8000 species, respectively [1]. In the growth and development, environmental adaptation, and quality formation of various biological processes in horticultural plants, gene transcription has long been regarded as a key regulatory mechanism [2]. Signaling components, including transcription factors, have been extensively documented to play critical roles in the development and stress adaptation of horticultural plants. However, due to its rapid and precise control over target proteins, post-translational modification (PTM) has emerged as an important modulator in these processes.

SUMOylation, which involves the covalent attachment of small ubiquitin-like modifier proteins (SUMO) to target proteins via the formation of isopeptide bonds, represents a crucial form of PTM in eukaryotic cells [3]. SUMO shares structural similarities with ubiquitin but possesses a distinct sequence. The mature SUMO molecules are produced from their precursors through cleavage by SUMO proteases. Like ubiquitination, SUMOylation encompasses three key steps: activation, conjugation, and ligation [4]. Initially, the mature SUMO protein is activated by a SUMO E1 activating enzyme complex before being transferred to a SUMO E2 conjugating enzyme. Typically aided by SUMO E3 ligases, SUMO is then covalently linked to specific lysine residues on target proteins [3]. In certain instances, poly-SUMO chains may be generated on protein substrates through the catalytic action of SUMO E4 ligases. SUMOylation is also a reversible process, with de-SUMOylation being mediated by SUMO proteases [5]. In addition, many proteins contain a SUMO-Interacting Motif (SIM), which facilitates the noncovalent interaction between these proteins and SUMO molecules [6].

Given that SUMO conjugations play a crucial role in influencing the stability, trafficking, interactions, and activity of target proteins [7], they may potentially regulate biological processes associated with these substrate proteins in plant cells. Proteomics studies have demonstrated that thousands of proteins serve as substrates for SUMOylation in Arabidopsis [8, 9], and the regulatory mechanisms and molecular roles of this PTM have been extensively investigated in this model plant [10]. However, the mechanisms and functions of SUMOylation in horticultural plants remain poorly understood. Specifically, research in this field has primarily focused on several species, such as apple (*Malus domestica*), tomato (*Solanum lycopersicum*), and pepper (*Capsicum annuum*). Thus, the present summary and perspective aim to provide valuable insights to guide future studies in other horticultural crops. Although SUMOylation is a conserved critical process in eukaryotic cells, its molecular roles vary among species. Therefore, it is believed that research on the specificity of SUMOylation functions in horticultural plants could significantly enhance our understanding of regulatory mechanisms and contribute to advancements in molecular breeding for horticultural crops.

## The SUMO system in horticultural plants

SUMOylation is facilitated by a conserved enzymatic cascade in eukaryotic cells, and various SUMO isoforms may be utilized in this process. However, the number of these enzymes and SUMO molecules varies across plant species, and functional specificity may also differ among homologs [5]. Recently, the components of the SUMOylation system have been identified in several horticultural plants (Fig. 1).

**Figure 1:**
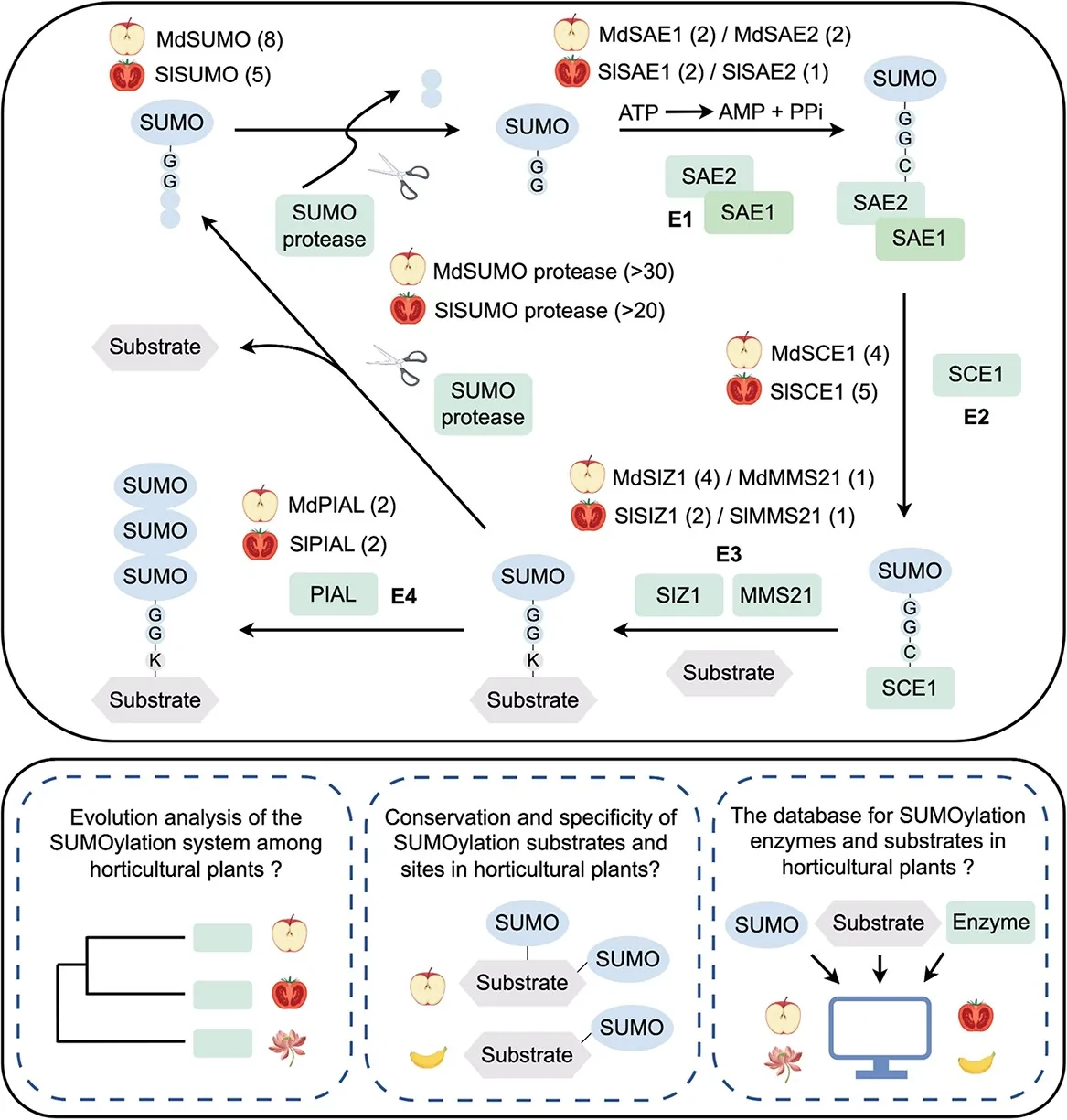
The SUMOylation system in horticultural plants. The process of SUMOylation is illustrated. The numbers of SUMO isoforms, E1 (SAE1; SAE2), E2 (SCE1), E3 (SIZ1; MMS21), E4 (PIAL), and SUMO proteases in apple and tomato are depicted in the upper graph. The unresolved questions and future directions for investigating the SUMO system in horticultural plants are outlined in the lower graph. ‘G’ represents glycine residue; ‘C’ represents cysteine residue; and ‘K’ represents lysine residue.

Eight SUMO genes were identified in Arabidopsis through bioinformatics analysis, but only the expressions of *AtSUMO1*, *AtSUMO2*, *AtSUMO3*, and *AtSUMO5* are detectable, suggesting that these four SUMO molecules play predominant roles [11]. Similarly, eight SUMO genes exist in apple, with *MdSUMO1* and *MdSUMO3* exhibiting high expression levels across all tissues [12]. In tomato (*S. lycopersicum*), five SUMO isoforms have been identified [5]. Given that different target proteins may be specifically modified by distinct SUMO members, the diversity of SUMO isoforms may contribute to the conjugation specificity of substrate proteins in horticultural plants. However, further systematic biochemical analyses are required to provide supporting evidence for this hypothesis.

The E1 SUMO-activating enzyme complex is composed of the regulatory subunit SAE1 and the catalytic subunit SAE2. In Arabidopsis, there are two isoforms of SAE1 and one member of SAE2 [11], and the depletion of E1 genes results in embryo lethality [13], thus underscoring the unique role of this complex in SUMOylation. Similarly, tomato possesses two *SAE1* genes and one *SAE2* gene [5]. In contrast, apple has been found to contain two copies of both the *SAE1* and *SAE2* genes [12]. However, the functions of the SUMO E1 members in horticultural plants remain to be investigated. In Arabidopsis, only a single SUMO E2 conjugating enzyme, AtSCE1, has been characterized, and the depletion of this gene also results in embryo lethality [13]. Notably, four SCE1 isoforms are present in apple and five in tomato [5, 12], suggesting a gene family expansion in horticultural plants. Additionally, the expression levels of several *SCE* genes are significantly upregulated in response to various abiotic stresses in potato [14]. This diversity may provide opportunities for functional redundancy or specificity among these enzyme isoforms in horticultural plants.

In Arabidopsis, two SUMO E3 ligases have been identified: AtSIZ1 and AtMMS21. AtSIZ1 predominantly plays a role in various stress responses [15], while AtMMS21 is primarily associated with developmental processes [16]. Given that the biological functions of SUMO E3 ligases are substrate-dependent, the roles of their homologs may vary across different horticultural plant species. In contrast to ubiquitin ligases, which possess numerous members that confer substrate specificity, the specificity of SUMO ligases is considerably lower. Similarly, two homologs of SIZ1 and one homolog of MMS21 were identified in tomato [5]. Notably, while one member of MMS21 was found, four members of SIZ1 were detected in apple [12], indicating a functional diversity among SUMO ligases. The higher number of SIZ1 homologs in apple suggests that enhanced regulatory complexity in developmental processes and environmental adaptation may represent a potential evolutionary driver of gene expansion in perennial horticultural plants. Functional studies demonstrated that the SIZ1 homologs from tomato, apple, pepper, broccoli, and sweet potato play significant roles in various processes [17–21]. Similarly to Arabidopsis [22], two SUMO E4 ligases (PIALs) have been identified in tomato [5], but their function remains unclear.

Compared to SUMO ligases, Arabidopsis exhibits a significantly higher number of families and members of SUMO proteases; for instance, ESD4, FUG1, ELS1/2, SPF1/2, OTS1/2, and DESI proteases have been identified [23]. This observation supports the hypothesis that the regulation of specificity for SUMOylated proteins may be contributed by de-SUMOylation processes mediated by SUMO proteases. Recently, it has been reported that the pepper SUMO protease CaDeSI2 plays a crucial role in regulating drought response through its mediation of de-SUMOylation [24]. More than 20 SUMO proteases in tomato and over 30 SUMO proteases in apple were predicted, suggesting that this family of enzymes may play a significant role in the de-SUMOylation of various protein substrates.

Therefore, the SUMOylation system in horticultural plants exhibits a more complex mechanism that may play specific functions in regulating proteins through SUMOylation. In future studies, it would be interesting to analyze the evolution of SUMOylation systems among horticultural plants. Modification-specific proteomics may uncover the conservation and specificity of SUMOylation substrates and their modified sites across different species. The compilation of these data would contribute to the establishment of a comprehensive database for SUMOylation enzymes and substrates in horticultural plants.

## SUMOylation in the development of horticultural plants

SUMOylation has been demonstrated to play significant roles in various plant developmental processes [10]. For example, both of the two SUMO ligase genes in Arabidopsis are involved in development regulation [25–27], but AtSIZ1 regulates growth predominantly dependent on salicylic acid signaling [28], whereas AtMMS21 controls the root phenotype via modulating the cell cycle and DNA repair [29]. In contrast, in horticultural plants, several SIZ1 homologs have been shown to be involved in regulating different developmental processes; however, the role of MMS21 homologs remains unclear in this context.

First, SUMOylation modulates key signaling components involved in the regulation of organ development in horticultural plants. MdCNR8, which plays a crucial role in regulating organ size during fruit and root development, has been identified as an interactor with MdSCE1 and as a substrate for SUMOylation mediated by MdSIZ1. SUMOylation facilitates the translocation of MdCNR8 from the plasma membrane to the nucleus. Furthermore, overexpression of *MdSIZ1* significantly enhances root development in apple, thereby supporting its function in positively regulating cell proliferation [30]. Therefore, precise regulation of subcellular localization through SUMOylation constitutes an important strategy in horticultural plants. In addition to its role in primary root development, SUMOylation is also involved in the regulation of lateral roots in apple. The auxin response factor MdARF8 interacts with the SUMO E2 enzyme MdSCE1 and has been identified as a substrate for SUMOylation by MdSIZ1. MdSIZ1 enhances the enzymatic activity of MdSCE1, facilitating the formation of a complex comprising MdSCE1, MdSIZ1, and MdARF8 that promotes the SUMOylation of MdARF8. Importantly, SUMOylation is crucial for maintaining the protein stability of MdARF8, indicating a functional interplay between SUMOylation and ubiquitination. Furthermore, overexpression of either *MdARF8* or *MdSIZ1* significantly stimulates lateral root formation in apple plants, thereby underscoring their respective roles in this developmental process [31]. Interestingly, the Arabidopsis auxin response factor ARF7 also undergoes SUMOylation to regulate root branching patterns in response to water availability, but this modification attenuates the DNA-binding activity of ARF7 [32], suggesting that SUMOylation-mediated regulation of ARFs operates through distinct mechanisms across different plant species.

The regulatory mechanism mediated by SUMOylation, which similarly influences auxin signaling, also plays a role in the abscission process of tomato. The transcription factor SlGATA6 positively regulates the expression of genes such as SlIAA3, which is essential for maintaining the gradient of auxin response necessary for the SUMOylation of the auxin response factor SlARF2a, a key suppressor of auxin signaling and initiator of abscission. Furthermore, both SlIAA3 and its homolog SlIAA32 interact and inhibit the function of SUMOylated SlARF2a, thereby attenuating its activity. Consequently, SUMOylation precisely modulates auxin signaling during abscission via modulating the interaction of ARF and IAA factors in tomato [33]. Collectively, these findings from apple and tomato establish an auxin response network that is regulated by SUMOylation in horticultural plants.

SUMOylation may also contribute to the development of gametophytes in strawberries (*Fragaria viridis*). The overexpression of FviBAG6-A in strawberries results in seed abortion and a reduction in seed number, with this protein interacting with FviSIZ1 to facilitate its SUMOylation [34]. However, further evidence is required to establish the direct function of SUMOylation in this process. Interestingly, AtBAG7, a member of the BAG family found in Arabidopsis, undergoes SUMOylation in the endoplasmic reticulum before translocating to the nucleus for gene transcription regulation during stress responses [35]. Although studies have demonstrated that SUMOylation is crucial for normal meiosis and gametophyte development in both Arabidopsis and maize [36, 37], the regulatory mechanism of SUMOylation in this process may differ in horticultural plants. In sunflower (*Helianthus annuus*), two heat stress transcription factors, HaHSFA9 and HaHSFA4a, have been identified as substrates for SUMOylation. Given that HaHSFA9 and HaHSFA4a play crucial roles in seed longevity, their SUMOylation may contribute to embryogenesis and seed development in sunflowers [38]. Therefore, it would be important to elucidate the molecular role of SUMOylation in the development of gametophytes and seeds in horticultural plants in future studies.

Additionally, SUMOylation is involved in regulating metabolism during the development of horticultural plants. MdSCE1, the SUMO E2 enzyme in apple, interacts with MdMYB30, a transcription factor that governs cuticle wax accumulation via regulating the transcription of genes such as *MdKCS1*. Furthermore, MdSIZ1 facilitates the SUMOylation of MdMYB30, thereby protecting it from ubiquitination-mediated degradation by the 26S proteasome. Consequently, the MdSIZ1-dependent SUMOylation positively influences wax accumulation and cuticle permeability in apple [39]. Similarly, the SUMO E4 ligase MdPIAL2 plays a crucial role in the deposition of wax on apple leaves by stabilizing the transcription factor MdHDG5. In contrast, the ubiquitin E3 ligase MdMIEL1 facilitates the degradation of both MdHDG5 and MdPIAL2, thereby negatively regulating leaf wax deposition [40]. Therefore, SUMOylation targets key transcription factors to prevent their degradation for modulating gene transcription in metabolism during the development of horticultural plants. In previous proteomics studies, numerous proteins associated with metabolism, including transcription factors and metabolic enzymes, have been identified as substrates for SUMOylation in plant cells [8], indicating that this modification may play a critical role in diverse plant metabolic processes.

Collectively, SUMOylation is involved in the precise regulation of various developmental processes in horticultural plants, including organ size control, lateral root development, abscission, gametophyte formation, and cuticle wax accumulation (Fig. 2). In future studies, it will be essential to analyze the profiles of SUMOylation enzymes and substrates across various tissues and developmental stages in horticultural plants. Previous research has primarily focused on the function of SUMOylation in auxin signaling, but its involvement in regulating other hormones such as brassinosteroids (BRs), cytokinins (CKs), and gibberellins (GAs) in development, as well as the interactions among these hormones, warrants further investigation. Given that metabolites significantly influence the quality of products derived from horticultural crops, it is worthwhile to investigate the function of SUMOylation in modulating their metabolic pathways.

**Figure 2:**
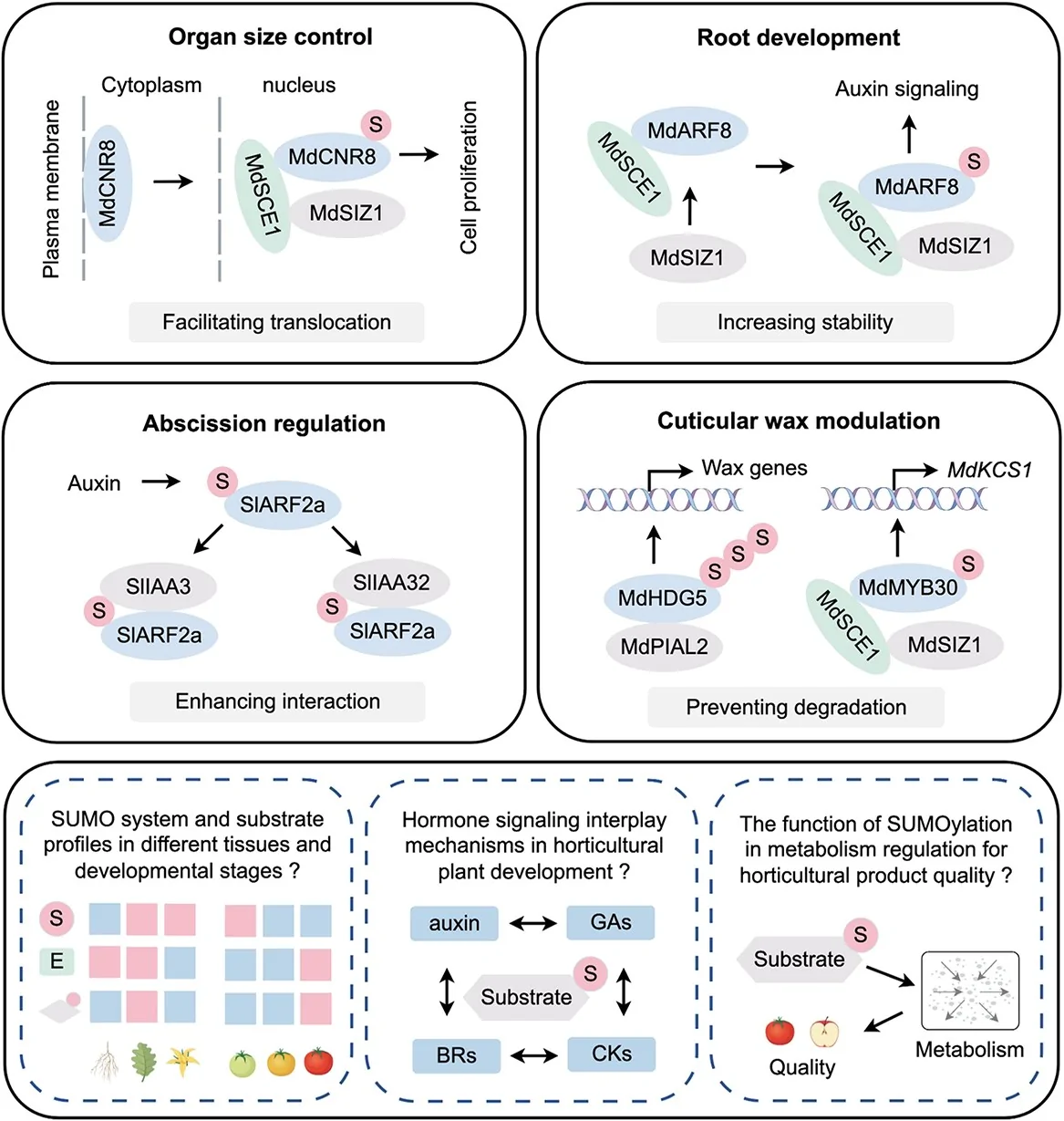
SUMOylation in the development of horticultural plants. The roles of SUMOylation in regulating developmental processes of horticultural plants are illustrated in the upper graph. MdSIZ1 and MdSCE1 mediate the SUMOylation of MdCNR8, thus facilitating its nuclear translocation to promote cell proliferation during organ size regulation. SUMOylation mediated by MdSIZ1 and MdSCE1 maintains the protein stability of MdARF8 in root development. SlIAA3 and SlIAA32 interact to inhibit the activity of SUMOylated SlARF2a for regulating auxin signaling during abscission. Both MdSIZ1-mediated SUMOylation of MdMYB30 and MdPIAL2-mediated SUMOylation of MdHDG5 enhance their stability to promote cuticular wax-related gene expression. The remaining questions and future perspectives regarding this topic are summarized in the lower graph. ‘S’ represents SUMO; ‘E’ represents SUMO-associated enzymes.

## SUMOylation in stress responses in horticultural plants

In eukaryotic cells, SUMOylation is consistently upregulated in response to various stresses, establishing this modification as a general protective mechanism for cells against environmental challenges. SUMO conjugations may create a hub for target proteins, enabling them to maintain their structure and function under stressful conditions [3]. For example, general protein SUMOylation is significantly enhanced during heat stress and this process is mediated by AtSIZ1 [41]. Global SUMOylation substrates and SUMO-associated chromatin regions have been identified to elucidate the role of this AtSIZ1-dependent modification during the heat stress response [9, 42]. Although SUMOylation is also involved in the response to various stresses, including cold, heat, drought, nutrient deficiency, and pathogen attack, in horticultural plants, the underlying molecular mechanisms exhibit specific features distinct from those characterized in Arabidopsis.

MdSIZ1 acts as a central regulator in the cold response of apple, with its expression being upregulated under low-temperature conditions. A transcription factor, MdMYB2, binds to the promoter region of *MdSIZ1* to activate its expression during stressful situations. Consistently, the expression levels of both *MdSIZ1* and *MdMYB2* contribute to enhanced cold tolerance [43]. Additionally, another transcription factor, MdMYB1, an essential regulator in anthocyanin biosynthesis, has been identified as an interacting partner of MdSIZ1. MdSIZ1 facilitates the SUMOylation of MdMYB1, thereby enhancing its protein stability for anthocyanin production under low-temperature conditions [44]. Furthermore, MdCIbHLH1, a cold-induced transcription factor, is also a substrate for SUMOylation mediated by MdSIZ1. As an ICE1-like protein, MdCIbHLH1 binds to the promoter of *MdCBF2* to activate its transcription in response to cold stress [45]. Notably, SUMOylation also enhances AtICE1’s protein stability by suppressing ubiquitination and modulating the transcriptional activity of its target genes during cold responses [46]. Therefore, SUMOylation serves as a critical regulatory mechanism by targeting key transcription factors involved in plant responses to low temperatures across plant species.

In addition to its role in cold response, SUMOylation is also implicated in heat stress responses in horticultural plants. Global SUMO conjugations are significantly upregulated under high-temperature conditions in tomato. Overexpression of *SlSIZ1* enhances tolerance to heat stress, whereas knockdown of *SlSIZ1* results in increased sensitivity to heat in tomatoes. SlSIZ1 interacts with SlHSFA1 and facilitates its SUMOylation, thereby regulating its activity and promoting the accumulation of heat stress proteins [47]. Similarly, AtHSFA2 also undergoes SUMOylation, which alters its activity in mediating the response to elevated temperatures [48]. Because heat shock transcription factors (HSFs) constitute a large family of transcription factors involved in stress responses, SUMOylation-dependent regulation of other HSF members in horticultural plants remains to be investigated. Additionally, AtDREB2A, another key transcription factor involved in heat stress responses in Arabidopsis, is a target protein for SUMOylation, which protects it from ubiquitination-dependent degradation [49]. Notably, MdDREB2A in apple also undergoes conjugations by MdSUMO2 that are upregulated under high temperatures. However, contrary to the mechanism observed in Arabidopsis, SUMOylation of MdDREB2A triggers its ubiquitination and subsequent degradation. The knockdown of *MdSUMO2* leads to an induced expression of heat-responsive genes and a higher survival rate among apple plants [50]. Consistently, under drought stress conditions, the conjugation of MdSUMO2 to MdDREB2A also promotes its degradation, a process that is mediated by the ubiquitin E3 ligase MdRNF4 [51]. Therefore, although SUMOylation targets DREB2A homologs both in apple and Arabidopsis species, it proceeds through different molecular mechanisms, highlighting the functional specificity of this modification among distinct plants.

In pepper, the transcription factor CaDRHB1 functions as a positive regulator in drought response and interacts with the SUMO ligase CaSIZ1. CaSIZ1 mediates the SUMOylation of CaDRHB1, which enhances its protein stability. Additionally, SUMOylation promotes the export of CaDRHB1 from the nucleus, leading to its accumulation in the cytoplasm [52]. Furthermore, knockdown of *CaSIZ1* increases the sensitivity of pepper plants to abscisic acid (ABA) and drought stress. Notably, CaSIZ1 also interacts with and facilitates the SUMOylation of CaABI5, a conserved transcription factor involved in ABA signaling transduction, indicating that this modification plays crucial roles in ABA-dependent stress responses [19]. Given that the process of SUMOylation is reversible, de-SUMOylation mediated by SUMO proteases also contributes to drought stress responses in pepper. The PP2C protein CaAITP1, which is involved in ABA signaling, is regulated through degradation mediated by ubiquitin ligase CaAIRE1. Under drought stress conditions, both SUMOylation and stability of CaAITP1 are diminished. Moreover, CaAITP1 interacts with a SUMO protease known as CaDeSI2 that promotes its de-SUMOylation. Knockdown of *CaDeSI2* results in increased accumulation of CaAITP1 and heightened sensitivity to drought [24]. Therefore, this dynamic regulation of target proteins via reversible SUMOylation establishes an intricate network for modulating drought responses in pepper.

The deficiency of essential nutrients represents a significant environmental stressor that adversely affects plant growth. MdSIZ1 is pivotal in regulating the responses to iron deficiency in apple plants. It directly targets the transcription factor MdbHLH104, which serves as a key component in modulating the plasma membrane H^+^-ATPase-mediated response to iron scarcity. The SUMOylation of MdbHLH104 mediated by MdSIZ1 is crucial for maintaining its protein stability. Consistently, the expression of *MdSIZ1* is upregulated under conditions of iron deficiency and positively influences iron uptake [53]. Similarly, the transcript level of *MdSIZ1* is also induced by phosphate deficiency, leading to a consequential upregulation of SUMOylation under these conditions. MdPHR1, the transcription factor associated with phosphorus starvation, serves as a target for SUMOylation, with a similar mechanism to its homolog in Arabidopsis [54]. The overexpression of *MdSIZ1* enhances the growth of apple callus in response to phosphate deficiency, indicating that SUMOylation plays a critical role in this stressful environment [55]. Future studies would benefit from exploring the function of SUMOylation in other nutritional deficiencies in horticultural plants.

Besides abiotic stress responses, SUMOylation is implicated in the interactions between pathogens and horticultural plants. Geminiviruses represent a large family of DNA viruses that infect various plant species, including many horticultural crops. The viral DNA replicates within the plant nucleus by utilizing host machinery, such as PCNA, a conserved factor involved in DNA replication [56]. Notably, Rep, a viral protein essential for replication from the representative geminivirus Tomato yellow leaf curl virus (TYLCV), induces specific alterations in the SUMOylation patterns of host plants. SlPCNA from tomato undergoes SUMOylation at two lysine residues; in animal and yeast cells, this modification enhances the activity of PCNA homologs involved in DNA metabolic processes. Rep interacts with SlPCNA to interfere with its SUMOylation, potentially affecting the replication environment for both viral and host DNA [57]. Pathogenic bacteria employ an alternative strategy to modify the SUMOylation of host factors in horticultural plants. XopD, a secretion effector derived from *Xanthomonas euvesicatoria*, is essential for pathogen growth and symptom suppression in tomato leaves [58]. Importantly, XopD contains a SUMO protease domain and functions as a de-SUMOylating enzyme in plant cells. The tomato transcription factor SlERF4 plays a critical role in regulating ethylene production, which is a process necessary for immunity and symptom development. SlERF4 serves as a substrate for SUMOylation, but its SUMO conjugations are removed by XopD, leading to destabilization of SlERF4 and subsequent suppression of symptoms [59]. Another tomato transcription factor SlbHLH132, which regulates development and resistance, is also regulated by the de-SUMOylation mediated by XopD for its activation during the process of pathogenesis [60]. Therefore, in addition to endogenous plant SUMO proteases, pathogen-derived SUMO proteases may reprogram biological processes of horticultural plants through the de-SUMOylation of host proteins. In addition, a variety of SUMO system genes, along with SUMO conjugation processes, are upregulated in Caixin (*Brassica rapa* var. *parachinensis*) in response to the infection by the pathogenic bacterium *Pectobacterium carotovorum* [61]. In future research, it is essential to investigate the role of SUMOylation in the interactions between horticultural plants and other types of pathogens.

Collectively, SUMOylation plays a crucial role in the precise responses of horticultural plants to various abiotic and biotic stresses, including temperature fluctuations, drought conditions, nutrient deficiencies, and pathogen infections (Fig. 3). Recently, the identification of tissue specificity in the SUMO network has unveiled the precise regulatory mechanisms underlying stress responses in Arabidopsis roots [62]. Therefore, it will be essential to delineate the profiles of SUMOylation enzymes and substrates in response to various stress conditions in horticultural plants. Additionally, it is important to explore the roles of SUMOylation in the signaling interplay mechanisms of distinct hormones, such as ABA, ethylene, jasmonic acid (JA), and salicylic acid (SA), in the context of stress responses. Heat-induced SUMOylation targets different bacterial effectors, indicating a potential role of this PTM in modulating the interaction between temperature and pathogen responses [63]. Therefore, the functions of SUMOylation in mediating crosstalk between abiotic and biotic stress responses also warrant further investigation in horticultural plants. Continued research into these signaling interactions will be crucial for establishing a comprehensive network that integrates multiple stress response mechanisms in horticultural plants.

**Figure 3:**
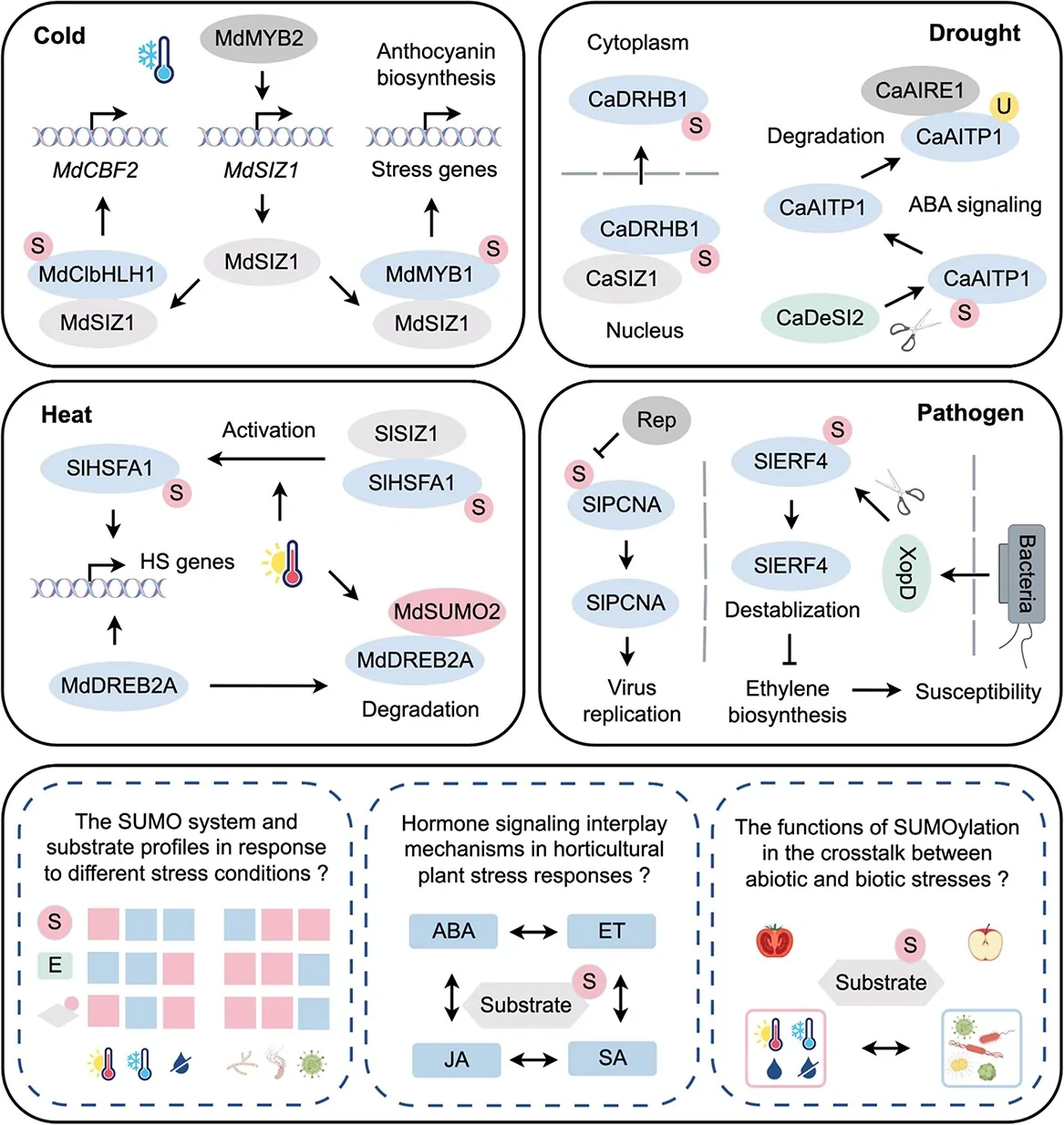
SUMOylation in stress responses in horticultural plants. The functions of SUMOylation in modulating the responses of horticultural plants to various stresses are illustrated in the upper graph. In the cold response, MdMYB2 activates the expression of MdSIZ1, which promotes the SUMOylation of MdMYB1 and MdCIbHLH1. In the heat stress response, SlSIZ1 enhances the SUMOylation of SlHSFA1 to regulate its activity, whereas MdSUMO2 targets MdDREB2A to trigger its ubiquitin-dependent degradation. In the drought response, CaSIZ1 SUMOylates CaDRHB1 to promote its cytoplasmic translocation and accumulation; conversely, CaDeSI2 de-SUMOylates CaAITP1, enabling its CaAIRE1-dependent ubiquitination and degradation. In the pathogen response, geminivirus Rep inhibits SlPCNA SUMOylation, while bacterial XopD de-SUMOylates SlERF4 to regulate ethylene biosynthesis. The unresolved issues and future directions regarding this topic are presented in the lower graph. ‘S’ represents SUMO; ‘U’ represents ubiquitin; and ‘E’ represents SUMO-associated enzyme.

## Potential functions of SUMOylation in hormone signaling in horticultural plants

Hormones, including auxin, ABA, ethylene, CKs, GAs, BRs, JAs, SA, strigolactones, and small peptides, play critical roles in various developmental processes and stress responses in plants [64]. In Arabidopsis, key components of most plant hormones have been reported to be regulated by SUMOylation [65]. Given the conserved mechanisms of hormone biosynthesis and signaling across plant species, it is believed that SUMOylation also plays a significant role in modulating hormone pathways in horticultural plants to regulate development and resistance processes. However, the roles of SUMOylation in regulating hormone signaling remain largely unexplored in horticultural plants; therefore, insights gained from model plant systems could inform and guide future investigations.

In horticultural plants, the role of SUMOylation in development has been reported to be closely associated with the auxin signaling pathway. Distinct members of the ARF transcription regulator family involved in auxin response, specifically MdARF8 from apple and SlARF2a from tomato, are directly regulated by SUMOylation [31, 33]. These mechanisms are different from the examples in Arabidopsis, supporting the specific modulation of auxin-related components via SUMOylation across various plant species. Given that IAA proteins form heterodimers with ARF proteins and that IAA17 has also been identified as a substrate for SUMOylation in Arabidopsis [66], it would be valuable to investigate whether IAA proteins are similarly modulated by SUMOylation during the development of horticultural plants.

Several studies have highlighted the crucial role of SUMOylation in regulating ABA signaling in horticultural plants. For instance, the expression levels of 12 genes involved in SUMOylation pathways in apple were significantly altered under ABA treatment [12], establishing a strong correlation between SUMOylation and ABA. Notably, the homologs of E3 ligase SIZ1 play essential roles in the ABA response across various horticultural plant species, including apple, tomato, pepper, broccoli, and dendrobium [17–20, 67]. For example, CaSIZ1 interacts with CaABI5, a transcription factor associated with ABA in pepper [19], but the detailed mechanism remains unclear. Consistently, AtABI5 is also targeted by AtSIZ1, and the SUMOylation of AtABI5 inhibits ABA signaling [68]. Furthermore, other components of ABA signaling such as MYB30 have been identified as substrates for SUMOylation in Arabidopsis [69]. Given that ABA is critical for responses to abiotic stressors, it would be beneficial to further characterize additional ABA signaling factors regulated by SUMOylation in horticultural plants.

In the ethylene signaling pathway, the tomato ethylene–responsive transcription factor SlERF4 is regulated by SUMOylation to modulate ethylene production [59]. Similarly, AtERF1, a member of this protein family from Arabidopsis, also undergoes SUMOylation to regulate photomorphogenesis [70]. In Arabidopsis, transcription factors associated with other plant hormone pathways are also subject to regulation by SUMOylation. For example, SUMOylation influences BZR1 in BR signaling [71], ARR1 in CK response [72], JAZ6 in JA pathway [73], and WRKY33 under SA conditions [74]. However, the specific components in these hormonal pathways that are regulated by SUMOylation remain to be identified in horticultural plants. Furthermore, small peptides represent an emerging class of plant hormones. A recent study has indicated that SUMO conjugation controls the processing of damage-associated peptides in Arabidopsis [75]. Consequently, there is a growing interest in investigating the regulatory mechanisms of SUMOylation concerning peptide hormones in horticultural plants.

Therefore, SUMOylation may be linked to distinct hormone signaling pathways in horticultural plants. It is essential to expand investigation into the roles of SUMOylation in regulating components involved in the signal transduction of hormones, such as BR, CK, JA, and SA. Importantly, the functional specificity of SUMOylation in regulating hormone signaling remains to be elucidated across horticultural and model plant species. Additionally, the role of SUMOylation in the crosstalk among various plant hormones in different biological processes is waiting to be explored in horticultural species.

## Potential methods for SUMOylation study in horticultural plants

The identification of protein substrates is a critical issue in the investigation of PTMs. However, proteomic approaches have rarely been employed to characterize SUMOylation target proteins in horticultural plants. A pioneering study conducted in 2017 aimed to identify SUMOylated proteins in potato (*S. tuberosum*). Total proteins were extracted from potato leaves for analysis using 3D electrophoretic gels, and the AtSUMO1 antibody was utilized to detect SUMO-modified proteins. Consequently, 39 individual proteins were identified as potential substrates for SUMOylation [76]. Building on this strategy, the changes in SUMOylation of potato protein substrates in response to infection by *Phytophthora infestans*, a significant pathogenic oomycete, were further characterized [77]. However, due to the specificity of the AtSUMO1 antibody and the resolution in protein electrophoresis, only a limited number of protein substrates were captured using this method. Unfortunately, subsequent efforts to identify SUMOylated proteins have lagged behind in horticultural plant studies, hindering deeper investigations into the molecular functions associated with this modification.

In recent years, several efficient methodologies have been developed to identify SUMOylation substrates in model plants [78], underscoring their potential applications in horticultural research. To analyze SUMOylated proteins in Arabidopsis, a 6 × His-AtSUMO1^H89R^ variant was employed to replace the endogenous AtSUMO1. When the SUMOylated proteins, enriched on Ni-NTA resin which captures the His tag, were digested with trypsin, the four C-terminal residues of this AtSUMO1 variant left a QTGG remnant at the modified site on target proteins, which can be characterized through mass spectrometric analysis [79]. Combined with quantitative proteomic methods, this strategy was utilized to investigate global changes in SUMO-conjugated targets in response to various types of stressors in Arabidopsis [80]. Recently, the K0 version of 6 × His-AtSUMO1^H89R^, where all seven lysine residues were substituted with arginine, was introduced into the *sumo1sumo2* mutant plants to enhance sensitivity for SUMOylation analysis. The Ni-NTA purified proteins were digested with Lys-C, a protease that cleaves at the C-terminal side of lysine residues, the intact 6 × His-AtSUMO1^H89R^(K0) would remain attached to SUMOylated peptides. These peptides could then be further enriched on Ni-NTA resins for sequential trypsin digestion and mass spectrometric characterization. Utilizing this method led to the identification of 2200 SUMOylation sites mapped to ~1300 potential protein substrates [8]. Consequently, these strategies provide valuable resources for globally analyzing SUMOylated proteins in horticultural plants, but the challenges with genetic transformation and antibody specificity need to be considered.

Because these methods require the introduction of 6 × His-AtSUMO1^H89R^ into *sumo1* mutant plants or 10 × His-AtSUMO1^H89R^(K0) into *sumo1sumo2* mutant plants [8, 79], their rapid application in horticultural studies may be challenging due to potential bottlenecks in efficient transformation and gene editing in horticultural plants. An alternative approach based on cDNA library offers a more straightforward method for identifying SUMOylation substrates in horticultural species. A Myc-tag cDNA library generated from specific plant species was transformed into bacterial cells, where the SUMOylation enzyme cascade was reconstituted. SUMOylated proteins in the total protein extracts from these bacterial cell mixtures were sequentially enriched using tags derived from 6 × His-FLAG-SUMO molecules and Myc-fused cDNA-encoded proteins. Following mass spectrometry analysis, 212 putative SUMOylated maize proteins were identified through this strategy [81]. Since this method does not need plant transformation or specific SUMO antibodies, it may facilitate the identification of SUMOylated proteins in horticultural plants, but the SUMOylation of candidate substrates must be further validated *in vivo*.

Another issue in SUMOylation studies is the characterization of enzymes responsible for specific protein substrates. In Arabidopsis, quantitative proteomics conducted using both wild-type and *AtSIZ1* mutant plants has revealed numerous protein substrates whose SUMOylation is mediated by AtSIZ1 [9]. Notably, while there are hundreds of ubiquitin ligases present in Arabidopsis, only a limited number of SUMO E3 ligases exist, indicating that the substrate specificity of SUMOylation may not be as pronounced as that of ubiquitination. Conversely, there are a higher number of SUMO proteases in plant cells [23], which supports the idea that the specificity of SUMO conjugations could be influenced by de-SUMOylation processes. Recently, a synthetic biological approach has been employed to identify SUMO proteases for protein substrates. In this method, distinct SUMO proteases (ubiquitin-like proteases; ULPs) are introduced into bacterial cells that have been reconstituted with the plant SUMOylation enzyme cascade, allowing for the rapid identification of de-SUMOylation enzymes corresponding to specific protein substrates [82]. This strategy does not require plant transformation, making it a powerful tool for studying the reversible SUMOylation mechanism in horticultural plants. However, the identified enzyme-substrate pairs derived from the bacterial system require further experimental validation in horticultural plants. Furthermore, for specific protein substrates, mass spectrometry analysis can be employed to efficiently identify multiple PTMs. This approach would facilitate further investigation into the functional interplay between SUMOylation and other types of modifications on a protein target.

Collectively, in future studies, approaches based on proteomics and synthetic biology can be utilized to identify SUMOylation protein substrates and enzymes in horticultural plants (Fig. 4). These technological advancements will significantly enhance the investigation of functions and mechanisms in this field.

**Figure 4:**
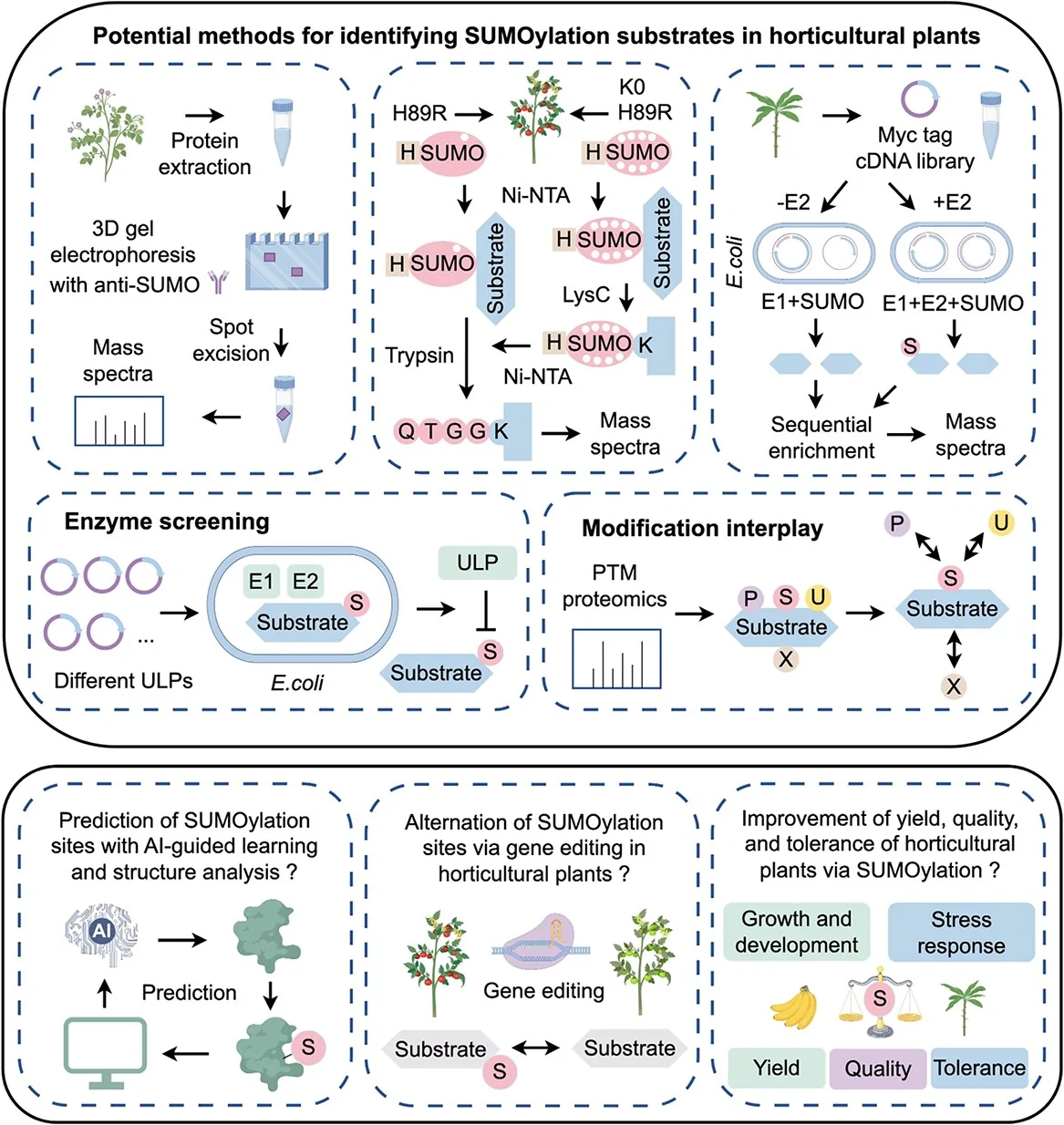
Potential methods and future perspectives for SUMOylation in horticultural plants. The strategies for identifying SUMOylation protein substrates, enzymes, and PTM interplay in horticultural plants are illustrated in the upper graph. The future perspectives of this field are depicted in the lower graph. ‘K’ represents lysine residue; ‘R’ represents arginine residue; ‘Q’ represents glutamine residue; ‘T’ represents threonine residue; ‘H’ represents the His tag; ‘S’ represents SUMO; ‘U’ represents ubiquitin; ‘P’ represents phosphorylation; and ‘X’ represents other PTMs.

## Conclusions and perspective on SUMOylation in horticultural plants

In summary, SUMOylation plays a significant role in modulating developmental processes and stress responses in horticultural plants (The SUMOylation substrates and their enzymes in horticultural plants are summarized in Table 1). These findings support the notion that SUMOylation is emerging as an essential mechanism for precisely controlling target proteins, thereby influencing corresponding signaling transduction in horticultural research. Therefore, further investigation into the remaining issues would be invaluable for researchers in this field. In future horticultural plant research, certain issues warrant particular attention; for instance, global identification of SUMOylation substrates, systematic analysis of the enzyme specificity, characterization of crosstalk between SUMOylation and other PTMs, and application of SUMOylation knowledge toward crop improvement.

**Table 1:** A summary of the function of SUMOylation in horticultural plants.

**Substrate protein**	**Species**	**SUMO component**	**Enzyme**	**Function**	**SUMOylation mechanism**	**Reference**
MdCNR8	Apple	E2; E3	MdSCE1; MdSIZ1	Organ size control	Mediating translocation	[30]
MdARF8	Apple	E2; E3	MdSCE1; MdSIZ1	Lateral root development; auxin signaling	Enhancing protein stability	[31]
SlARF2a	Tomato	Unknown	Unknown	Abscission regulation; auxin signaling	Facilitating protein interaction	[33]
FviBAG6-A	Strawberry	E3	FviSIZ1	Gametophyte development	Unknown	[34]
HaHSFA4a/9	Sunflower	Unknown	Unknown	Embryogenesis and seed development	Unknown	[38]
MdMYB30	Apple	E2/E3	MdSCE1/MdSIZ1	Wax accumulation and cuticle permeability	Enhancing protein stability	[39]
MdHDG5	Apple	E4	MdPIAL2	Leaf cuticular wax deposition	Enhancing protein stability	[40]
MdMYB1	Apple	E3	MdSIZ1	Cold stress response; anthocyanin biosynthesis	Enhancing protein stability	[44]
MdCIbHLH1	Apple	Unknown	Unknown	Cold stress response	Unknown	[45]
SlHSFA1	Tomato	E3	SlSIZ1	Heat stress response	Regulating activity	[47]
MdDREB2A	Apple	SUMO	MdSUMO2	Heat stress response; drought stress response	Promoting degradation	[49, 51]
CaDRHB1	Pepper	E3	CaSIZ1	ABA and drought stress response	Mediating translocation; enhancing protein stability	[52]
CaABI5	Pepper	E3	CaSIZ1	ABA and salt stress response	Unknown	[19]
CaAITP1	Pepper	SUMO protease	CaDeSI2	Drought stress response	Enhancing protein stability	[24]
MdbHLH104	Apple	E3	MdSIZ1	Iron deficiency response	Enhancing protein stability	[53]
MdPHR1	Apple	E3	MdSIZ1	Phosphate deficiency response	Unknown	[55]
SlPCNA	Tomato	Unknown	Unknown	Geminivirus resistance	Unknown	[57]
SlERF4	Tomato	SUMO proteases	XopD (*X. euvesicatoria*)	Bacteria resistance; ethylene signaling	Modulating protein stability	[59]
SlbHLH132	Tomato	SUMO proteases	XopD (*X. euvesicatoria*)	Bacteria resistance	Regulating activity	[60]

Given that SUMOylation systems are conservatively present in eukaryotic species, the characterization of SUMO components and substrates has thus far been limited to a select few horticultural plants, including apple, tomato, and pepper. More importantly, the roles of SUMOylation in regulating key processes central to horticultural biology, such as fruit ripening, flavor formation, perennial growth habits, and graft compatibility, remain largely unexplored. Further studies in these specific processes would uncover unique SUMOylation mechanisms in horticultural plants, distinct from those in Arabidopsis. With the genomes of numerous horticultural species now sequenced [83], there exists an opportunity to explore more comprehensively the factors associated with SUMOylation systems in additional horticultural crops. This includes investigating SUMO molecules, SUMO E1 activating enzymes, and SUMO conjugating enzymes, as well as SUMO E3 and E4 ligases and SUMO proteases across a wider array of horticultural plants. By integrating transcriptional data from various developmental stages and stress responses, it would be advantageous to compile this information into a comprehensive database for the study of SUMOylation in regulating development and stress responses in horticultural plants. Utilizing proteomics approaches [8, 79], global identification of SUMOylation substrates and their modification sites can be conducted across a broad spectrum of horticultural species, and these findings can also be incorporated into the established database. Consequently, this bioinformatics- and biochemistry-based database would serve as a valuable resource for further functional investigations into the role of SUMOylation in this field.

SUMOylation has been demonstrated to interact with other PTMs, such as ubiquitination and phosphorylation [84]. In horticultural studies, it has been shown that SUMOylation regulates the ubiquitin-dependent protein degradation in the modulation of development and stress responses. For instance, SUMOylation suppresses the degradation of MdMYB30 during cuticular wax regulation [39] but enhances the degradation of MdDREB2A in response to heat and drought stresses [49], highlighting distinct and opposing interplay mechanisms between SUMOylation and ubiquination across different target proteins. However, direct evidence for the crosstalk between SUMOylation and ubiquitination or other types of modifications, such as phosphorylation, remains to be further investigated. SUMOylation can occur at the same lysine residues to inhibit ubiquitination or be recognized by STUbLs to promote ubiquitination. Additionally, the SUMO attachment may enhance or attenuate the association between substrates and modifying enzymes, thereby modulating other PTMs such as phosphorylation. Based on these general rules, it would be important to analyze the functional interplay between SUMOylation and other PTMs in horticultural species using biochemical and genetic approaches. Additionally, since SUMOylation is involved in both development and stress responses [10], elucidating its function in the crosstalk between distinct biological processes is essential. For instance, abiotic stresses may induce the SUMOylation of certain substrates associated with developmental processes. Consequently, analyzing these interactions at either the biochemical or phenotypic levels will enhance our understanding of this modification in horticultural plants.

Using emerging technologies, SUMOylation can be more precisely controlled in horticultural plants (Fig. 4). Through artificial intelligence (AI)–supported structural analysis and learning from the established database [85], the prediction of SUMOylation sites on protein substrates may achieve greater accuracy. Furthermore, AI-mediated protein design has the potential to enhance enzyme activity and specificity during the SUMO conjugation process. Additionally, the SUMOylation sites on protein substrates in horticultural plants can be modified through gene editing targeting the codons corresponding to lysine residues. Reversibly, the incorporation of a specific motif may facilitate artificial SUMO conjugation on the target proteins. This strategy has the potential to facilitate the generation of novel horticultural germplasm resources. By employing these approaches, SUMOylation may be applied to modulate developmental processes and stress responses in horticultural plants, ultimately enhancing yield, quality, and resistance. Therefore, investigating the molecular functions and regulatory mechanisms of SUMOylation will deepen our understanding of PTMs across various biological processes and provide valuable insights into future genetic improvements in horticultural plants.
